# Congenital Microphthalmic Syndrome in a Swine

**DOI:** 10.1155/2018/2051350

**Published:** 2018-06-21

**Authors:** Radka Andrysikova, Titus Sydler, Dolf Kümmerlen, Wolfgang Pendl, Robert Graage, Romana Moutelikova, Jana Prodelalova, Katrin Voelter

**Affiliations:** ^1^Equine Department, Section of Ophthalmology, Vetsuisse Faculty, University of Zurich, Winterthurerstrasse 260, 8057 Zürich, Switzerland; ^2^Institute of Veterinary Pathology, Vetsuisse Faculty, University of Zürich, Winterthurerstrasse 270, 8057 Zürich, Switzerland; ^3^Department for Farm Animals, Division of Swine Medicine, Vetsuisse Faculty, University of Zürich, Winterthurerstrasse 270, 8057 Zürich, Switzerland; ^4^Veterinary Research Institute, Hudcova 296/70, 621 00 Brno, Czech Republic

## Abstract

A 17-week-old crossbred finishing pig was presented for lameness of approximately one week. Clinical evaluation, including ophthalmologic examination, revealed ataxia, partial flaccid paresis of the pelvic limbs, skin lesions at feet and claws, and severely reduced vision/blindness. Both eyes had multiple persistent pupillary membranes (iris-to-iris and iris-to-lens) and hypermature cataracts. Histopathological examination of the eyes revealed microphthalmia, microphakia with cataract formation, myovascularised membrane in the vitreous, retinal detachment, and retinal dysplasia. Microscopic examination of tissues collected postmortem demonstrated nonsuppurative polioencephalomyelitis with the most prominent inflammatory lesions in the lumbar spinal cord. Subsequently, presumed Teschen/Talfan disease was confirmed by porcine teschovirus identification in the spinal cord using the reverse transcription-polymerase chain reaction (RT-PCR). To the authors' knowledge, this is the first case report describing in detail histopathological changes in the porcine congenital microphthalmic syndrome.

## 1. Introduction

Several congenital ocular defects in pigs, such as anophthalmia, macro- and microphthalmia, and cyclopia, have been documented in the veterinary literature [1].

Microphthalmia, which is frequently associated with multiple malformation, has been described in both domestic and nondomestic species, including several canine breeds (e.g., Doberman Pinscher, Saint Bernard, and Miniature Schnauzer, but more frequently as part of the collie eye anomaly), horses, cattle (Angus, Shorthorn, Hereford), Texel sheep, pigs, camel, white-tailed deer, and raptors [2]. However, microphthalmia may be seen as sporadic finding in any species [3].

Microphthalmia syndromes may arise early in organogenesis caused by abnormal development of the optic vesicle (either deficiency in the optic vesicle or later because of retarded growth and expansion of the optic cup). Microphthalmia can also occur at later developmental stages as a result of insufficient early intraocular pressure [2]. If microphthalmia originates at the stage of the neural plate/optic sulcus, it is frequently associated with multiple ocular malformations (e.g., anterior segment dysgenesis, cataract, retinal dysplasia, and persistent hyaloid artery) [2]. Microscopic abnormalities typically include microphthalmos, opaque and pigmented cornea, aphakia or microphakia, and absence of a normal anterior chamber. Histological changes are represented by missing Descemet's membrane and/or normal corneal endothelium, disorganized cystic ciliary and iridal epithelium, and heterotopia (e.g., stratified squamous epithelium, glandular tissue, or cartilage in the anterior segment) [3].

A long-established cause of microphthalmia in swine is hypovitaminosis A [4–6]. Other known or suspected causes include gestational-acquired infections, drugs, and environmental pollutant toxicities [7, 8]. More recently, various genetic aberrations ranging from chromosomal duplications to mutations in a single gene* SOX2* [8] were identified to be implicated in microphthalmia development in humans and other animal species.

Teschen/Talfan disease is caused by the ubiquitous* Teschovirus A* (previously classified as Serogroup 1 porcine enterovirus), a nonenveloped ssRNA(+) virus in the order* Picornavirales*, family* Picornaviridae*. There are 13 serotypes (PTV-1 to -13) [9] of different pathogenicity, but all of them exhibit gastrointestinal and neurotropism. Both domestic pigs and wild boars are the only known hosts [10].

The disease manifests itself clinically as fever, diarrhea, and pneumonia, with subsequent paresis/paralysis of pelvic limbs. The most severely ill pigs may succumb to the disease after 3-4 days [11]. Chorioretinal lesions have been reported in infected pigs [1]. Histopathologically, necrosis, perivascular cuffing, neuronophagia, and gliosis are hallmarks of nonsuppurative polioencephalitis [10].

Teschen/Talfan disease has been documented in Central Europe, Denmark, Great Britain, Canada, USA, and Australia. It is a reportable disease in Switzerland as well as in other European countries under the Teschen Disease Order in domestic and European Union legislation [10]. Over the last decade, there have been reported 4-5 outbreaks per year in Switzerland (personal communication, Dr. Xaver Sidler).

This report documents the most complex case of porcine congenital microphthalmic syndrome to the authors' knowledge, characterized by microphthalmia, multiple persistent pupillary membranes (PPMs) which consisted of both iris-to-iris and iris-to-lens, microphakia with cataract formation, myovascularised membrane in the vitreous, retinal detachment, and retinal dysplasia.

## 2. Case Presentation

A 17-week-old Large White x Landrace crossbred fattening pig weighing 55 kg was presented to the Division of Swine Medicine, Vetsuisse Faculty, University of Zurich, for evaluation of lameness. The pig was housed on a finishing farm along with other 94 fattening pigs. The first symptom occurred a week prior to the consultation with eight animals affected with lameness (morbidity ca 8%). The initial treatment by the referring veterinarian consisting of Metacam (20 mg/ml meloxicam) and Betamox (150 mg/ml amoxicillin) failed to achieve improvement. Subsequently, the Swiss Pig Health Service (SGD) transported two finishing pigs to the Division of Swine Medicine of the Vetsuisse Faculty. One pig exhibited only ataxia and the other exhibited both ataxia and vision loss. The visual pig was not included in this report.

The blind pig was calm and mentally alert, but ataxic with partial flaccid paresis of the pelvic limbs, although able to stand up with help. There were several skin lesions of the feet and claws, with the joints within normal limits. Panniculus and interdigital reflexes were normal and the rectal temperature was 39.5°C. The pig appeared to be blind. It was able to urinate and defecate without problems.

**Figure 1:**
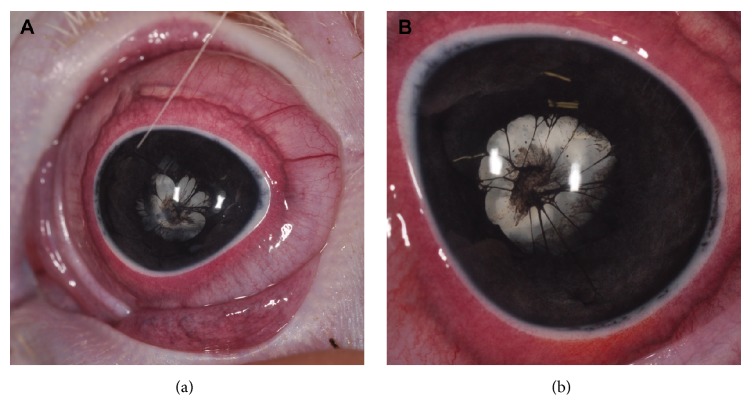
Macroscopic postmortem photograph of the left eye (a) and the right eye (b). Multiple persistent pupillary membranes (iris-to-iris and iris-to-lens) and hypermature cataracts are present in both eyes.

The ophthalmologic examination revealed normal palpebral and dazzle reflexes and negative pupillary light reflexes (PLR) in both eyes (OU). Vision was considered questionable based on reduced/questionable menace response OU. Examination with a portable slit-lamp biomicroscope (Kowa SL-15, Kowa Co., Ltd. Tokyo, Japan) revealed bilateral multiple PPMs (iris-to-iris and iris-to-lens) and bilateral hypermature cataracts (Figure 1). Adnexal structures and the rest of the anterior segment structures were normal OU. The posterior segment could not be examined due to the opacity of the optic media OU.

Because of the progressive worsening of neurologic signs, no treatment was attempted, and the pig was humanely euthanized and submitted to the Institute of Veterinary Pathology, Vetsuisse Faculty, University of Zurich, for pathological examination, which found no gross lesions in any of examined organ systems other than both eyes. Brain, spinal cord, and both globes were submitted for histopathological examination.

At necropsy, brain, spinal cord, and both globes were removed and fixed for several days in 10% buffered formalin, dehydrated in a descending alcohol series, then paraffin-embedded, cut in 2-3 *μ*m sections, mounted on glass slides, and stained using hematoxylin and eosin.

From the central nervous system, histological slides were prepared from cranial cortex with basal ganglia, cortex with hippocampus and thalamic regions, midbrain, cerebellum, pons, medulla oblongata, cervical, thoracic, and lumbar spinal cord. Craniospinal ganglia were not examined.

Histologically, a nonsuppurative polioencephalomyelitis was present from the hypothalamus to the lumbar spinal cord. However, concerning the grade of inflammatory cell contribution, the process must be declared as very mild in the brain, whereas inflammatory lesions were more prominent in the lumbar spinal cord. Additionally, the entire spinal cord exhibited a high grade of Wallerian degeneration. In diencephalic regions there were few vessels with few lymphocytes in the Virchow-Robin spaces and few small foci of intrameningeal lymphocytes present. In the mesencephalon few foci of lymphocytes were found in the meninges, few ones layered cuffed vessels and some degenerated neurons, showing vacuolation at the margin of the soma, and dilated myelin sheaths in the surrounding areas were visible. Cerebellum and its meninges were not affected and medulla oblongata showed sparse small glial nodules. Lesions in the spinal cord were much more prominent showing prominent glial nodules, perivascular cuffing, and neuron degeneration and mostly shrunken hypereosinophilic neurons in the grey matter and high grade Wallerian degeneration in the white matter. Wallerian degeneration was more prominent in the ventral spinal cord columns; however lateral and dorsal columns were also affected. Even in spinal roots Wallerian degeneration was present as well as few infiltrating lymphocytes. Unfortunately, spinal ganglia as well as trigeminal ganglia were not collected. Altogether, the histopathological picture was compatible with a subacute Teschen/Talfan disease condition.

**Figure 2:**
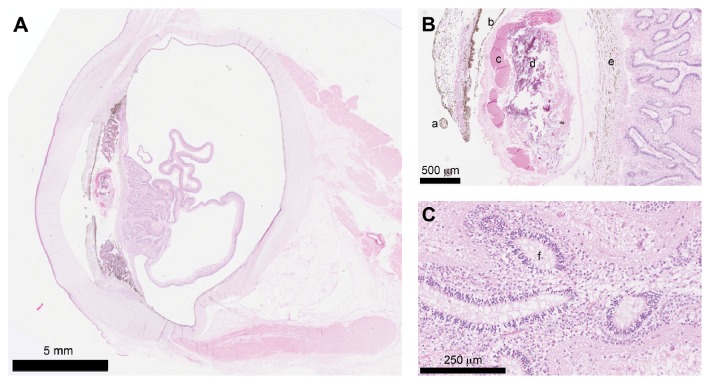
(A) Low power magnification image of the microphthalmic globe with microphakic cataractous lens and complete retinal detachment. Hematoxylin and eosin stain, bar = 5 mm. (B) High power magnification of persistent pupillary membrane (a), anterior portion of the myovascularised membrane (b), cataractous lens (c) with calcium crystals (d), and posterior portion of the myovascularised membrane (e). Hematoxylin and eosin stain, bar = 500 *μ*m. (C) High power magnification image of dysplastic retinal layers with rosettes (f) in the inner nuclear layer. Hematoxylin and eosin stain, bar = 250 *μ*m.

Ocular lesions included microphthalmia, multiple PPMs (iris-to-iris and iris-to-lens), microphakia with cataract formation, myovascularised membrane surrounding the cataractous lens and expanding into the vitreous towards the retina, retinal detachment, and retinal dysplasia (Figure 2). Moreover, Wallerian degeneration was identified in the optic nerve. In spite of the widespread ocular changes, the ciliary body was large and prominent. The detached retina contained only indistinctively/poorly developed different cellular layers; rosette formation was occasionally observed in the inner nuclear layer. These findings were classified as congenital microphthalmic syndrome. Since there was no histological evidence of inflammation in any of the examined ocular tissues, including the uveal tract and retina, the ocular changes were presumed to be of another cause than acute inflammatory disease.

Based on history, clinical examination, course of the disease, and histopathologically confirmed nonsuppurative polioencephalomyelitis, a tentative diagnosis of Teschen/Talfan disease was suspected and samples of brain and spinal cord were subjected to laboratory examination to test for the presence of the porcine teschovirus.

RNA extractions from both brain and spinal cord were performed using TRI Reagent® (Sigma Aldrich, St. Louis, MO, USA) according to the manufacturer's instructions. The negative control consisted of nuclease-free water (Top-Bio, Prague, Czech Republic) instead of biological material. Porcine teschovirus strain VIR 460/88 (CAPM V – 647) obtained from the Collection of Animal Pathogenic Microorganisms (CAPM, Veterinary Research Institute, Brno) was used as a positive control.

Isolated RNA was used for a one-step reverse transcription-polymerase chain reaction (RT-PCR) (Transcriptor One-step RT-PCR Kit, Roche Mannheim, Germany) using specific primers (PEV-1a-F: 5′-AGTTTTGGATTATCTTGTGCCC-3′, PEV-1e-R: 5′-CGCGACCCTGTCAGGCAGCAC--3′) targeting the highly conserved 5′-nontranslated region (5′-NTR) [12]. Used primers generated 316 bp product characteristic to members of the genera* Teschovirus*. Finally, to further increase specificity of our PCR reaction second “nested” PCR was performed (Aptamer Hot Start Master Mix, Top-Bio, Prague, Czech Republic) (158 bp, PEV-1c: 5′-TGAAAGACCTGCTCTGGCGCGAG-3′, PEV-1d: 5′-GCTGGTGGGCCCCAGAGAAATCTC-3′) [12]. Both obtained amplicons were sequenced commercially (SeqMe, Příbram, Czech Republic) and data were analyzed using BLAST and aligned with GenBank sequences.

Porcine teschovirus was detected in spinal cord, but not in the brain sample. Comparison of acquired 316 bp sequence from 5′-NTR locus with teschovirus sequences available in GenBank showed 96% nucleotide similarity with porcine teschovirus strain BL7792 (GenBank no. GQ293236).

## 3. Discussion

Among congenital globe abnormalities reported in pigs, such as anophthalmia, macro- and microphthalmia, cyclopia, and retinal dysplasia, microphthalmia is the leading ocular anomaly [1, 7]. The most frequent cause of the congenital globe abnormalities is maternal vitamin A deficiency. Insufficient levels of vitamin A in farm animals have been reported in populations with limited access to natural sources of vitamin A and inadequate or no vitamin supplements [6, 13–16]. If hypovitaminosis A resolves before the 12th day of gestation, no malformation in newborn piglets is observed and eye organogenesis is normal in piglets when dams receive vitamin A supplement at earlier stages of pregnancy [17]. On the contrary, if malnutrition persists up to day 90, abnormalities of spinal cord, brain, skin, and tail arise [18]. These changes are well documented in piglets born to vitamin A-deficient dams receiving subnormal doses of vitamin A until at least 18 days after conception. In the same study it was found that mesenchymal tissue (e.g., iris and primary vitreous body) and not ectoderm was the primary target of the hypovitaminosis A. Failed closure of the fetal optic fissure, abnormal mesenchymal proliferation anterior and posterior to the lens, and decreased rate of globe growth during the last two-thirds of pregnancy were hallmarks in piglets of vitamin A-deficient sows [17]. However, ocular tissue development observed in the case reported here, as well as lack of other hallmarks associated with early to mid-gestation hypovitaminosis A (e.g., spinal cord, brain, skin, and tail abnormalities), suggests that either hypovitaminosis A in late gestation period or other teratogenic insults were the main causes of microphthalmia.

Apart from hypovitaminosis A, several other causes of microphthalmia were reported, including suspected drug toxicities and high levels of selenium in diet [7]. Hereditary microphthalmos has been reported in Yorkshire pigs [1], White Shorthorn cattle, Jersey calves, and Hereford cattle (as a part of encephalopathy-microphthalmos syndrome). The pattern of heritability seems to be autosomal recessive trait in all cases listed above [7].

The globes evaluated in this case had PPM strands. Persistent pupillary membranes represent remnants of the vessels and mesenchyme of the* tunica vasculosa lentis anterior* (so called pupillary membrane) in the developing eye. This primitive vascular system reaches maximal development by day 45 in the dog, then begins to regress, as the aqueous humor takes over this metabolic function, [2] and completely disappears in dogs within the first weeks after the birth [2, 3, 19]. Pupillary membrane,* tunica vasculosa lentis*, and hyaloid artery undergo first apoptosis, later cellular necrosis, until complete atrophy [2]. When these mechanisms fail, PPMs, persistent hyperplastic primary vitreous (PHPV), and persistent hyperplastic* tunica vasculosa lentis* (PHTVL) remain [20]. The normal age of pupillary membrane's regression in swine is not known [1].

Although rarely reported in swine, PPMs are reported occasionally in dogs and horses, and inheritability has been recognized in several canine breeds [20].

One report documented a very high frequency of PPMs occurrence in Yucatan micropigs reaching 66.1% of micropigs between 7 and 12 months of age. Similar to the case reported in this study, other associated pathologies including presence of hyaloid remnants, congenital cataracts, nuclear opacities, and suture line abnormalities were diagnosed in up to 80% of examined Yucatan micropigs [21].

Lenticular abnormalities, such as cataract or microphakia, are common hallmarks of the anterior segment dysgenesis. Although congenital cataracts are found in many species, only few of them are linked to maternal exposure to toxins, infection (e.g., rubella virus in humans), or other* in utero* insults during the critical stages of lenticular development [3]. Congenital perinuclear (at the nuclear/cortical junction) cataracts developed in piglets whose mothers had been treated with alloxan [1]. Juvenile cataract, which is believed to be inherited, has been described in Vietnamese pot-bellied pigs [1].

The retinal abnormalities (retinal dysplasia with occasional rosettes) observed in our fattening pig represent the disorganized development of retinal tissue that may be accompanied by pigment disturbance or evidence of associated retinal degeneration [22].

Retinal dysplasia is an important hereditary abnormality in many breeds of dogs [2] but is seldom encountered as a genetic disease in other species [3]. Retinal dysplasia may be associated with maternal infection, e.g., infection with bovine viral diarrhea–mucosal disease (BVD-MD) virus (pestivirus) later than 76 days but before 150 days of gestation or intrauterine infection with feline panleukopenia virus among others [3].

In the swine described in this case report, the exact cause of the clinically apparent and histologically confirmed congenital microphthalmic syndrome was not determined. Since there were no other reported instances of microphthalmia in the swine herd or siblings of the affected pig, hypovitaminosis A seems to be less likely cause of microphthalmia in this case. Moreover, other symptoms observed in the animal (e.g., multiple PPM and retinal detachment) are usually not linked to hypovitaminosis A. On the contrary, other characteristic symptoms of hypovitaminosis A, including abnormalities of brain, spinal cord, tail, and skin, were absent in the examined pig. Sporadic genetic mutation and maternal exposure to toxic or infectious Noxa were considered to be more likely etiology. The histological changes in the central nervous system and presence of porcine teschovirus detected in spinal cord by the RT-PCR, while the brain sample was assessed negative, confirmed previous Teschen/Talfan infection, explaining the clinical symptoms, for which the finishing pig had been originally presented to the Division of Swine Medicine of the Vetsuisse Faculty, University of Zurich. There were no apparent inflammatory changes, neither in retina nor in choroid; therefore the authors assume that the ocular pathology was solely caused by a harmful event* in utero* in the last third of gestation (based on the retinal stratification) and the later infection had no (or minimal) influence on these changes.

In conclusion, we present a case of complex microphthalmic syndrome in pig, involving structural changes of anterior chamber (multiple iris-to-iris and iris-to-lens PPMs), lens (microphakia, hypermature cataract), and posterior segment (vitreal myovascularised membrane, retinal dysplasia with occasional rosettes in the inner nuclear layer, and retinal detachment). The probable etiology seems to be either sporadic genetic mutation or maternal exposure to toxic compound or infection.
